# Anatomy of diagnosis in a clinical encounter: how clinicians discuss uncertainty with patients

**DOI:** 10.1186/s12875-022-01767-y

**Published:** 2022-06-17

**Authors:** Maram Khazen, Erin E. Sullivan, Jason Ramos, Maria Mirica, Mark Linzer, Gordon D. Schiff, Andrew P. J. Olson

**Affiliations:** 1 Harvard Medical School and Brigham and Women’s Hospital, Boston, USA; 2 Sawyer School of Business, Suffolk University, Boston, USA; 3 Department of Global Health and Social Medicine, Harvard Medical School, Boston, MA USA; 4 Emory University School of Medicine, Atlanta, USA; 5 Brigham and Women’s Hospital, Boston, USA; 6 Department of Medicine at Hennepin Healthcare, University of Minnesota, Minneapolis, MN USA; 7 Division of General Internal Medicine, Brigham and Women’s Hospital, Boston, USA; 8 University of Minnesota Medical School, Minneapolis, USA

**Keywords:** Diagnostic uncertainty, Diagnostic utterances, Clinical reasoning, Qualitative methods, Primary care

## Abstract

**Background:**

Studies consider the clinical encounter as linear, comprising six phases (opening, problem presentation, history-taking, physical examination, diagnosis, treatment and closing). This study utilizes formal conversation analysis to explore patient-physician interactions and understanding diagnostic utterances during these phases.

**Methods:**

This study is a qualitative sub-analysis that explores how the diagnosis process, along with diagnostic uncertainty, are addressed during 28 urgent care visits. We analyzed physicians’ hypothesis-generation process by focusing on: location of diagnostic utterances during the encounter; whether certain/uncertain diagnostic utterances were revised throughout the encounter; and how physicians tested their hypothesis-generation and managed uncertainty. We recruited 7 primary care physicians (PCPs) and their 28 patients from Brigham and Women’s Hospital (BWH) in 3 urgent care settings. Encounters were audiotaped, transcribed, and coded using NVivo12 qualitative data analysis software. Data were analyzed inductively and deductively, using formal content and conversation analysis.

**Results:**

We identified 62 diagnostic communication utterances in 12 different clinical situations. In most (24/28, 86%) encounters, the diagnosis process was initiated before the diagnosis phase (57% during history taking and 64% during physical examination). In 17 encounters (61%), a distinct diagnosis phase was not observed. Findings show that the diagnosis process is nonlinear in two ways. First, nonlinearity was observed when diagnostic utterances occurred throughout the encounter, with the six encounter phases overlapping, integrating elements of one phase with another. Second, nonlinearity was noted with respect to the resolution of diagnostic uncertainty, with physicians acknowledging uncertainty when explaining their diagnostic reasoning, even during brief encounters.

**Conclusions:**

Diagnosis is often more interactive and nonlinear, and expressions of diagnostic assessments can occur at any point during an encounter, allowing more flexible and potentially more patient-centered communication. These findings are relevant for physicians’ training programs and helping clinicians improve their communication skills in managing uncertain diagnoses.

## Background

One of the most important activities and outcomes of a clinical encounter is diagnosis – the process by which clinicians and patients collaborate to identify the cause of the patient’s symptoms to inform management and prognosis [1]. Both the diagnostic process (diagnosis as a verb) and diagnostic outcomes (diagnosis as noun) have substantial room for improvement, given the unacceptably high burden of diagnostic error-associated harms in modern healthcare [2]. However, there is a paucity of studies qualitatively analyzing medical encounters through a diagnostic safety and improvement lens, especially considering the new National Academy of Medicine (NAM) definition of diagnosis quality that stresses “communication of the diagnosis to the patient;” communication is an essential aspect of good diagnosis [3]. Thus, improving diagnostic processes and outcomes requires better understanding of the modern medical encounter.

The structure and flow of the acute care visit has been traditionally conceptualized as comprising six linear phases: the opening, problem presentation, history-taking, physical examination, diagnosis and, treatment plan and closing [4, 5]. However, the process of clinical reasoning - the gathering and analysis of clinical information and deciding about diagnosis, prognosis, and treatment - is often not linear and involves non-analytic decision-making by clinicians; diagnostic hypotheses are often generated very early in encounters and, likewise, clinicians make many diagnoses by pattern recognition rather than hypothetico-deductive reasoning [6]. Although many researchers agree that the diagnostic phase is where the clinician shifts from gathering information to delivering it [7], many argue that the diagnosis process occurs at different times during the encounter as preliminary, speculative, or hypothetical [8].

Given that a key aspect of the encounter is communicating about the diagnostic assessment with patients, it is valuable to move beyond simply informing patients of “the diagnoses” to more meaningfully engaging patients as co-equal members of the diagnostic team [9, 10]. While many patients may expect diagnoses to be delivered at the end of a clinical encounter - and medical education curricula teach to such expectations - this “diagnosis as product” paradigm betrays the underlying, non-linear process suggested by the modern diagnostic reasoning literature [11, 12]. Additionally, this more stereotyped expectation of giving a diagnosis at the end of the encounter is setting-related and may be more the norm in acute/urgent care compared to general chronic care or annual medical visits, where a visit may be more focused on prevention and management rather than diagnosis [13].

A final important construct is diagnostic uncertainty, which in recent years has become a focus of attention in better understanding diagnosis and diagnosis-related communication. Diagnostic uncertainty is both ubiquitous and poorly understood, particularly in terms of how it plays out in clinical encounters [14]. Recent studies show that clinicians negotiate diagnostic uncertainty indirectly rather than explicity [15, 16] in order to safeguard against diagnostic errors without compromising their authority, credibility and ability to reassure anxious patients [17].

This study is part of a larger study, MD-SOS [Measuring Diagnosis: Safety or Stress], funded by Harvard’s medical malpractice insurer, which aims to link the diagnostic process with work conditions, stress, and burnout. The larger MD-SOS study aims to examine clinical notes and the actual encounters in relation to prominent diagnostic elements (e.g., addressing chief concern, differential diagnosis, uncertainty, red flags, time frames, contingencies, pejorative language) and triangulate what occurs during the encounter to the level of clinicians’ burnout, as suggested in previous work [18]. This sub-analysis aims to capture and analyze diagnostic communication and uncertainty communicated by the physician during the urgent care visit. We aimed to understand the timing of diagnostic reasoning and how that relates to the resolution of uncertainty by real-world physicians. We examined the degree to which diagnostic assessments were organized in linear vs. non-linear fashion and ways uncertainty was expressed.

## Methods

### Study design

We conducted an analysis of the clinical encounter MD-SOS transcripts. This descriptive and qualitative analysis builds upon previous studies [19], using the art of conversation analytic to explore patient-physician interactions and better understand the structure of conversations during clinical encounters [20, 21]. For this analysis, we defined diagnostic utterances as the spoken words which involved a synthesis of clinical and investigative data aimed at generating a diagnosis. We inductively examined how diagnosis and diagnostic uncertainty were addressed during urgent care visits by (a) identifying and categorizing diagnostic utterances throughout the six traditional encounter phases; (b) noting revisions of certain/uncertain diagnostic utterances during the encounter; and (c) examining how physicians tested their diagnostic hypothesis and managed uncertainty during the communication of diagnostic information. This project received approval from the Institutional Review Board at Mass General Brigham. We used COREQ guidelines for reporting qualitative research.

### Setting, participants, and recruitment

Study participants were primary care physicians (PCPs) and their patients, recruited from Brigham and Women’s Hospital (BWH) at three different urgent care settings in the greater Boston area. To increase generalizability, two sites were affiliated with a primary care clinic and one site was a walk-in urgent care center affiliated with emergency department. The principal investigator approached PCPs to invite participation and those who were willing to participate provided written consent. PCPs were offered a $75 gift card for their participation.

Research staff approached patients of enrolled physicians in the clinic waiting room, introduced them to the study and provided written information explaining that the encounter would be recorded and reviewed. Patients were given sufficient time to read the written information and refer to the research staff for questions. To minimize interruptions to the workflow, PCPs asked for a verbal consent from patients who agreed to participate, once they were in the room. The verbal consent was recorded as part of the encounter, as approved by the ethics committee.

### Procedures/data collection

Before beginning the actual clinical encounter, the physician obtained verbal consent from the patient to record the visit. Two research staff members were available on site to answer any questions after the visit but were not present in the exam room during the encounter (i.e., only the digital recorder was in the room). At the end of each clinic session, recorded speech files from the encounters were collected by the research assistant and stored in a secure password-protected file area and then transcribed using Amazon Transcribe, then further de-identified and edited by the research assistant for accuracy.

### Data analysis

Data analysis was completed by two researchers: a PhD communication expert and a PhD qualitative methods expert. Transcripts were entered into NVivo12 qualitative data analysis software and data were analyzed inductively and deductively, using content and conversation analysis [22–24]. With inductive analysis, the researchers allowed the themes to emerge from the data, while with the deductive analysis, the researchers applied the concepts from the MD-SOS clinical note tool (developed using accepted diagnostic elements) to generate themes by which to code the data [25]. Using an iterative process, the two researchers independently coded each transcript, coming together to reconcile coding decisions. The researchers reached 100% agreement on coding decisions after each review and coding reconciliation meeting.

The entire MD-SOS project team also was consulted during bi-weekly meetings to ensure consistent application of the codes and to identify emerging issues or disagreements discovered in adapting the clinical note tool to analyze the encounter transcripts. To acknowledge and minimize the influence of researcher bias on the data, the encounter data was triangulated with the clinical note, and findings were presented, discussed, and revised during team meetings. To enhance validity, codes and themes were reviewed throughout the coding process with reference to recordings to avoid the loss of paralinguistic information affecting meaning (e.g., hesitant voice tone).

### Analyzing the diagnostic process

For this analysis, we focused on the hypothesis generation process by highlighting three main codes: the location of diagnostic utterances during the encounter, whether certain/uncertain diagnostic utterances were expressed and revised throughout the course of the encounter, and how physicians tested their hypothesis generation and managed diagnostic uncertainty.

#### Location of diagnostic utterances

Each diagnostic utterance was categorized according to when it appeared with respect to one of the six classic phases of the encounter (opening, problem presentation, history-taking, physical examination, diagnosis, and treatment plan and closing). We defined the first diagnostic utterance and the beginning of hypothesis generation according to the first time a diagnosis was suggested after patients described their symptoms and the reason for their visit. Time stamped digital recordings and encounter transcripts assisted in identifying each phase of the encounter. Phrases that indicated the chief concern phase included: “what brings you in today”, “tell me the story/what’s going on”, “you’re here because …”, “how can I help you today?”

After patients had described their symptoms (problem presentation phase), the physician began the history taking phase, which included specific inquiries about red flags such as, “have you had a fever, how high has your fever been?” or expressions such as “and the thing that worries you the most right now…?” or “how did it start?”

Expressions that signaled the beginning of the physical exam were physicians’ expressions “let’s have a look”, “let me examine you to see what your [lungs or heart] sound like”, “I’m going to take a look and a listen,” or, specific sentences indicating an examination: “take a nice deep breath”, “Can you open your mouth?”,” I’m going to feel your head and neck”.

The end of the physical examination phase and beginning of the diagnosis phase was typically indicated by expressions such as: “come back and have a seat”, or “whenever you’re ready, you can sit up” or when the physician went back to checking the medical record.

The beginning of the diagnosis phase was established when the physical examination phase ended, and the diagnostic explanation and treatment plan was identified. A diagnostic utterance after the physical exam and before the treatment plan and closing was coded as the diagnosis phase. In this phase, physicians often provided leaflets or guidelines.

#### Revising diagnostic hypotheses

In addition to identifying the location of diagnostic utterances, we examined whether expressions of diagnostic uncertainty were revised to more certain expressions, if certain expressions were revised to uncertain expressions, or whether expressions remained the same throughout the encounter. We categorized diagnostic utterances as certain or uncertain. Diagnostic certainty was associated with sentences with assertive and probabilistic words and expressions (e.g., definitely”, “certainly,” “I am sure/certain,” “probably,”), and diagnostic uncertainty was identified according to words implying uncertainty (e.g., “suspect,” “I think,” “not sure,”, “possible”, “inclined,” “I can’t say 100%”).

## Results

Seven attending physicians practicing in 3 different urgent care settings (2 urban, 1 suburban) affiliated with Brigham and Women’s Hospital (BWH) participated in the study. Between January and March 2020, a total of 42 patients were approached and asked to participate; 14 declined (67% participation rate) while 28 agreed to have their encounters recorded. During the study, the clinical encounters ranged from 15 to 45 minutes. Given the relatively small sample, to ensure confidentiality of participating physicians and their patients, we did not collect identifying characteristics.

In most (24/28, 86%) encounters, the diagnosis process was initiated before the classic diagnosis phase and in some cases the diagnosis was discussed again during the treatment plan and closing. We identified 62 diagnostic communication utterances in 12 different clinical situations (ophthalmic infection, sinusitis, gastroenteritis, dyspepsia, inflamed toe, upper/lower respiratory infection, late menstrual cycle, headaches, weight loss, fall, abdominal pain, strained ankle/wrest, ear pain) within our data set.

A categorization of when the first diagnostic consideration was expressed by the physician is presented in Table 1. In addition to the initial diagnostic utterance, most encounters contained 1–2 additional utterances. In 17 encounters (61%), the diagnosis phase was not observed. Further, diagnostic utterances during the history-taking phase were accompanied by a second utterance within the visit, either during the diagnosis phase or treatment plan. This suggests that when the generation of a diagnostic hypothesis started earlier in the encounter, it was sometimes associated with additional diagnostic utterances.

**Table 1:** Location of diagnostic utterances and the thinking process according to the 6 encounter phases

Encounter	Opening	Problem Presentation	History Taking	Physical Examination	Diagnosis	Treatment & Closing
1			1st			2nd
2			1st, 2nd			3rd
3			1st, 2nd			3rd
4				1st		3rd
5				1st, 2nd		3rd
6				1st	2nd	
7				1st		2nd
8				1st	2nd	3rd
9				1st		2nd
10					1st	2nd
11					1st	2nd
12			1st		2nd	3rd
13			1st		2nd	3rd
14			1st, 2nd	3rd		
15			1st	2nd	3rd	
16				1st		2nd
17			1st	2nd		
18			1st			2nd
19			1st	2nd	3rd	
20					1st	
21				1st	2nd	
22				1st		
23				1st	2nd	
24				1st	2nd	
25					1st	
26				1st		2nd
27				1st	2nd	
28					1st	2nd

### Acknowledging uncertainty

In the data set, we observed physicians frequently acknowledging diagnostic uncertainty, an act less frequently reported in the literature (Table 1). For example, during history taking, a physician described uncertain aspects of diabetes in the following manner: “I don’t think we know exactly how much losing more weight or taking more sugars out of your diet, will prevent diabetes.” In another case, a patient inquired whether they had the flu. The physician responded during the physical examination: “I would think is that this could be the flu. And you can get the flu. Even though you’ve got the flu shot.” In some encounters, uncertain diagnostic hypotheses remained uncertain, or unresolved, at the treatment and closing phase.

### Revising and actively prioritizing a Diagnosis

During the hypothesis generation process, diagnostic uncertainty that was expressed early in the encounter became more certain later in 10 encounters (36%). For example, during history taking, a patient complained of a painful swollen toe and the physician said: “I think most likely this is not an infection, that it’s gout. “However, in the treatment and closing, the diagnostic utterance was certain: “The fact that you had the same thing a couple months before [in] that same toe and how it looks right now, where it’s really just the toe that’s red and tender, that sounds like gout and that’s where most of the pain was.”

Similarly, in a case of a suspected virus, the physician maintained during the physical examination that: “Your uvula is a little inflamed which would make me think this is more viral.” Whereas, during the diagnosis phase the diagnostic utterance became more certain: “It’s definitely not pneumonia. I think this is probably some other kind of virus, not flu.”

Additionally, a physician during the physical exam discussed the likelihood of a suspected stye, telling the patient, “I think you have a blockage of one of the glands on the upper lid there, that’s causing the inflammation. You’re getting some drainage as well.” The physician was asked by the patient during the diagnosis phase: “So what would you say? Conjunctivitis or a stye? Or both?” and the physician answered: “It’s a stye. It’s primarily a stye, and you have some drainage from that.”

These examples provide insights on the hypothesis generation process in the early parts of the encounter that become verified as the encounter moves forward into the traditionally described diagnosis or treatment plan phases. However, in other cases, physicians had an uncertain diagnosis in one phase, ruled out a diagnosis during the physical exam, and then revised it again to uncertain during treatment and closing. One example is a patient experiencing tightness in the throat, where during history taking, the physician said: “Something else that could be a sign that you’re not aware of is acid. It gets up to your throat and at your larynx so irritates it and tickles. So that’s always high on my list when someone kind of has an odd feeling in the throat.” Then during the physical exam, the physician said, “You certainly don’t have strep throat or something like that.” Finally, at treatment and closing the physician concluded: “I do suspect that you probably have some reflux.”

#### Testing the hypothesis generation process

When communicating diagnostic reasoning, the diagnostic uncertainty was managed either by requesting tests, or at times explicitly avoiding ordering requested tests that the physician felt were unnecessary tests (tests that would not substantively contribute to the diagnostic process). When physicians ordered testing, they communicated to patients that the test might not add additional information.

### Managing uncertainty expressions by ordering tests

Physicians often ordered tests when a diagnosis was uncertain. For example, during the treatment plan in a case of unidentified ear pain, a physician explained:

We don't see an obvious reason for this ear pain. It kind of sounds more like a nerve pain. Given some of your other vague complaints, the neck pain and the shoulder pain, I think we should do a little bit of a workup. Just do an EKG and check some routine blood work to make sure everything looks okay.

Also, during a sprained ankle case, a physician was hesitant during physical examination to order an x-ray: “It doesn’t really look like a fracture to me. It really seems like a little bit more like some of these ligaments. So, I’m wondering what is right. I’m not really sure what’s going to show us. I … feel that an orthopedic examination was probably going to be the next best thing for us. And the question is, do I do an ultrasound?” The encounter ended with the physician ordering an ultrasound: “So you’re going to get an ultrasound today… I’m pretty sure it’s going to be negative, but I think it’s just one of those things.”

Another example provided by a physician who ordered an x-ray to manage uncertain diagnosis of a strain or a fracture: “So I think we need an x-ray of your foot, because I am concerned that you may have a break. You have point tenderness in that one spot. And that’s kind of a common area to get a break. Let’s wait for the x-ray. I could still be wrong and it’s just a bad strain.”

### Managing uncertainty by safeguarding against ordering unnecessary tests

In other cases, physicians resolved or managed uncertainty while avoiding further tests. It appears that, in such cases, physicians explained their reasoning more explicitly, ensuring that while uncertainty was still present, there was not significant risk contained therein. In some cases, physicians detailed differential diagnoses as a way of explaining why imaging was not needed. For example, a physician explained to a patient experiencing a severe headache during the treatment plan: “Your headache symptoms have multiple possible causes. There is some suggestion of a tension headache. There is also a migrainous component to your headache. Finally, your eyes may be contributing to your headaches.” By using this explicit reasoning and differential diagnosis, the physician elucidated why an MRI was not necessary at that point.

Focusing on risks when communicating uncertain diagnosis during the treatment plan and closing phase was used in one encounter to avoid ordering potentially unnecessary (or even harmful) tests:

I think this is probably a little colitis or gastroenteritis. I can't say 100% (it) is not your appendix, but it's less likely because you don't typically get diarrhea. I mean, it's possible, like anything possible appendicitis is, it's common. Everyone has an appendix and the odds of something like 10 to 15% of people at some point in their life will get appendicitis.

The physician concluded during treatment and closing: “it seems like at this point to go against my judgement and subject you to radiation... just isn’t worth it, because of a very small likelihood of appendicitis.” Thus, the patient did not receive an x-ray or ultrasound.

## Discussion

Until recently, the clinical encounter was characterized in more stereotyped linear events segmented into more compartmentalized defined parts [4, 5]. Nonetheless, in modern acute care clinical encounters, clinicians are expected to share and engage the patient in clinical reasoning, although this remains an aspirational goal. The present study shows that the process of diagnosis generation in actual clinical encounters is nonlinear in two ways. First, nonlinearity was observed when diagnostic utterances occurred throughout the encounter (something our team termed “diagnosis on the fly”) and delivering the diagnosis occurred jointly with presenting treatment options. This is not dissimilar to prior studies referring to diagnostic utterances during “history taking” and the physical examination parts of the visit as hypothesis generation and verification [26]. Our findings suggest that the six phases of the encounter (opening, problem presentation, history-taking, physical examination, diagnosis, treatment plan and closing) should be viewed as overlapping and interrelated, integrating elements of one phase with another. Second, nonlinearity was also noted with respect to the communication and resolution of uncertainty, or lack of it: uncertainty diagnostic utterances were sometimes revised to certain during the encounter, while other times more uncertainty was added as the encounter went on.

Further, in this study, actively addressing diagnostic uncertainty was a frequent and integral component of the urgent care clinical encounter. The findings show that the process of communicating diagnostic information almost always included uncertainty expressions. This implies that sharing uncertainty may be more common than reported, a noteworthy finding especially in this time-pressured acute care setting. In our study uncertainty expressions can be depicted as a communication strategy and a way to explain diagnostic reasoning to patients, something suggested in previous studies [27, 28].

It appears that clinicians’ uncertainty is generally manifested by explicitly sharing their differential diagnosis with patients. Differential diagnosis is a fundamental activity in clinical reasoning [29] and making this process explicit for patients appears to be effective in explaining why the diagnosis could be one of several possibilities, or why or why not further testing and/or follow-up is needed. While it has been noted that such differential diagnoses are often missing from written encounter notes (which has proved particularly bedeviling in defending malpractice diagnosis error allegations [30, 31]), our findings raise the possibility that encounter transcripts may reveal more conscientious assessments than those documented in the medical record. Further, Physicians in this study managed and acknowledged uncertainty, while jointly creating contingency plans of returning to the clinic if symptoms worsen. This could be interpreted as a way of reassuring patients and partnering with them when delivering the diagnosis. Uncertain diagnostic hypotheses were tested either by ordering tests or they may be left unresolved, owing to explicit efforts to avoid ordering and subjecting patients to unnecessary or harmful tests. In several cases we noted that clinicians would acquiesce to ordering a less costly or harmful test than one with more risk (i.e., ordering an ultrasound rather than a CT scan). Further, we noted that uncertainty was discussed when both choosing to order tests to resolve that uncertainty or when consciously not ordering tests. It appears that test ordering thresholds and behaviors center on uncertainty related to risk of the condition as well as of the test [32, 33] itself.

This study focused on the US context and style of physicians in addressing diagnostic uncertainty. We did not inquire about physicians’ perceptions of malpractice concerns in relation to discussing diagnostic uncertainty. However, it is possible that physicians who managed uncertainty by ordering tests, did so due to malpractice concerns. While, this corresponds with literature concerning practicing defensive medicine [34, 35], a deeper understanding of what guided uncertainty discussions, could be explored in future studies.

It would be interesting in future studies to examine the frequency of discussing risk – of both the diagnosis and the diagnostic process - with patients.

Scholars have sought practical ways to embrace uncertainty in healthcare, looking for changes made in the system infrastructure, such as diagnostic codes and treatment algorithms and tips or tools to help clinicians manage uncertainty [36–38]. However, in our study, physicians seemed to readily acknowledge and embrace uncertainty, often indicating to patients that there is no single clear “right answer” when they explained their diagnostic reasoning. Clinicians, thus, appear to discuss uncertainty naturally, though indirectly [14, 15], even during relatively brief, focused encounters.

**Fig. 1:**
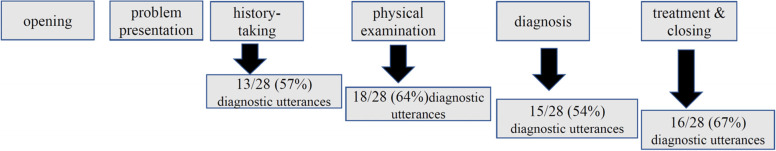
Interactive and Nonlinear Process of Diagnosis

Our findings have implications for programs aiming at improving clinicians’ communication skill in managing uncertain diagnosis as well as for programs teaching about clinical encounters and diagnostic reasoning. While a linear model of a clinical encounter may be a helpful introductory tool, it would be good for students to be taught that diagnosis is often more interactive and nonlinear and that expressions of diagnosis can occur at any point, allowing more dynamic, interactive, and flexible patient-centered communication (Fig. 1).

The findings point to several areas for future study. One is to further explore the types of diagnostic utterances and expressions of uncertainties as recorded in actual clinical encounters as we have done, with a larger sample of physicians and encounters. A second area for study is to understand how diagnostic evaluation and communication behaviors vary across different physicians, diagnoses, and settings. Third, it would also be important to examine the calibration and impact of uncertainty with different patients’ health literacy capabilities. If uncertainty is stated, is it truly understood comparably by all patients, and does it increase or decrease understanding, trust, and patient-desired reassurance?

### Limitations

Participants were selected through a convenience sample. To recruit a diverse sample, we recruited from three different clinics, and approached all patients in the waiting room without prior knowledge of their clinical condition. Since our study was limited to the greater Boston area and there is little data regarding discussing diagnostic uncertainty and its variations within the US, future studies could include clinics from different regions. The data was collected in early 2020, prior to the COVID-19 upsurge; our plans to recruit a larger sample size were interrupted by restrictions and risks imposed by the pandemic and thus we were unable to expand data collection beyond our initial 28 patients. Further, it is possible that because physicians knew they were recorded, they changed behaviors and provided more thorough explanations than they would in unrecorded encounters. However, physicians were blinded to any research hypotheses, and most stated that they were not consciously thinking about the recorder being turned on during the encounters.

## Conclusion

This study shows the evolution of diagnostic assessment utterances starts in the early stages of the encounter and often evolves with second or third utterances as the ultimate diagnostic statement(s) that occur toward the end of the encounter. The findings may be useful to educational and clinical programs promoting patient-centered care and partnering with the patient when communicating an uncertain diagnosis, specifically programs focusing on how patients process and utilize evolving diagnostic information transmitted by the clinician. As we move toward more meaningful co-production of diagnosis between patients and clinicians, a better understanding of the diagnostic process in the real world, including how diagnostic uncertainty is addressed and managed, is an important step.

## Data Availability

The datasets of the current study are available from the corresponding author on reasonable request.

## Abbreviations

PCP: primary care physicians
MD-SOS: Measuring Diagnosis: Safety or Stress
